# Nano-Silica Bubbled Structure Based Durable and Flexible Superhydrophobic Electrospun Nanofibrous Membrane for Extensive Functional Applications

**DOI:** 10.3390/nano13071146

**Published:** 2023-03-23

**Authors:** Misbah Batool, Hasan B. Albargi, Adnan Ahmad, Zahid Sarwar, Zubair Khaliq, Muhammad Bilal Qadir, Salman Noshear Arshad, Rizwan Tahir, Sultan Ali, Mohammed Jalalah, Muhammad Irfan, Farid A. Harraz

**Affiliations:** 1 Department of Chemistry, University of Sargodha, Sargodha 40100, Pakistan; misbah.batool095@gmail.com; 2 Promising Centre for Sensors and Electronic Devices (PCSED), Advanced Materials and Nano-Research Centre, Najran University, Najran 11001, Saudi Arabia; hbalbargi@nu.edu.sa (H.B.A.); msjalalah@nu.edu.sa (M.J.); 3 Department of Physics, Faculty of Science and Arts, Najran University, Najran 11001, Saudi Arabia; 4 Department of Textile Engineering, National Textile University, Faisalabad 37610, Pakistan; zahidsarwar@ntu.edu.pk (Z.S.); tahirizwan@gmail.com (R.T.); sultan@ntu.edu.pk (S.A.); 5 Department of Materials, National Textile University, Faisalabad 37610, Pakistan; zubntu@yahoo.com; 6 Department of Chemistry and Chemical Engineering, Lahore University of Management Sciences, Lahore 54792, Pakistan; salman.arshad@lums.edu.pk; 7 Electrical Engineering Department, College of Engineering, Najran University, Najran 61441, Saudi Arabia; miditta@nu.edu.sa; 8 Department of Chemistry, Faculty of Science and Arts at Sharurah, Najran University, Sharurah 68342, Saudi Arabia

**Keywords:** superhydrophobicity, nano-silica, nano-roughness, composite electrospun fibers, self-cleaning, water–oil separation, membranes, robustness

## Abstract

Nanoscale surface roughness has conventionally been induced by using complicated approaches; however, the homogeneity of superhydrophobic surface and hazardous pollutants continue to have existing challenges that require a solution. As a prospective solution, a novel bubbled-structured silica nanoparticle (SiO_2_) decorated electrospun polyurethane (PU) nanofibrous membrane (SiO_2_@PU-NFs) was prepared through a synchronized electrospinning and electrospraying process. The SiO_2_@PU-NFs nanofibrous membrane exhibited a nanoscale hierarchical surface roughness, attributed to excellent superhydrophobicity. The SiO_2_@PU-NFs membrane had an optimized fiber diameter of 394 ± 105 nm and was fabricated with a 25 kV applied voltage, 18% PU concentration, 20 cm spinning distance, and 6% SiO_2_ nanoparticles. The resulting membrane exhibited a water contact angle of 155.23°. Moreover, the developed membrane attributed excellent mechanical properties (14.22 MPa tensile modulus, 134.5% elongation, and 57.12 kPa hydrostatic pressure). The composite nanofibrous membrane also offered good breathability characteristics (with an air permeability of 70.63 mm/s and a water vapor permeability of 4167 g/m^2^/day). In addition, the proposed composite nanofibrous membrane showed a significant water/oil separation efficiency of 99.98, 99.97, and 99.98% against the water/xylene, water/n-hexane, and water/toluene mixers. When exposed to severe mechanical stresses and chemicals, the composite nanofibrous membrane sustained its superhydrophobic quality (WCA greater than 155.23°) up to 50 abrasion, bending, and stretching cycles. Consequently, this composite structure could be a good alternative for various functional applications.

## 1. Introduction

In recent times, superhydrophobic membranes are receiving much interest in various emerging applications [1,2,3,4]. Additionally, the poor adherence of water with the superhydrophobic surfaces causes the water drops to roll, resulting in surface cleaning by microorganisms [5]. Generally, the rough hierarchical architecture can impart a superhydrophobic character due to the minimum liquid/solid interface, low surface energy, and trapped air in surface pores [6]. A water contact angle (WCA) greater than 150° can be achieved by inducing nanoscale surface roughness [7]. Various techniques, including plasma surface modification [8,9], phase separation [10,11], the sol–gel approach [12,13], surface modification [14,15], and electrospinning [16,17] have been employed to induce micro/nano surface roughness [18,19]. These techniques provided nano-level cutting-edge hierarchical structures for designing textured surfaces [20,21,22]. Among these approaches, electrospinning is a flexible, cost-effective method for developing nanofibrous membranes. The electrospun nanofibrous membranes exhibited superhydrophobicity and adjustable porosity architectures [23,24,25,26,27].

Along with high water resistance ability, the electrospun superhydrophobic membranes also offered highly micro- and nano-porous surfaces, contributing to breathability and permeability for non-aqueous solvents [28]. Owing to these exceptional characteristics, superhydrophobic membranes have potential applications for water/oil separation [29,30,31] and personal protective equipment [32]. Moreover, integrating organic and inorganic nanoparticles/nanofibers on the nanofibrous membrane surface can impart various characteristics and superhydrophobicity [33]. Therefore, superhydrophobic nanofibrous membranes, developed through the surface coating of these nanostructures, can be utilized for multimodal applications, such as self-cleaning [34] and water purification [35].

Polyurethane (PU) is a potential polymer with exceptional stretch and recovery properties that offer hydrophobic characteristics. PU has been used in various research works with other materials that offer hydrophobic properties [36,37]. The roughness of the polymer surfaces can be increased by incorporating NPs, leading to superhydrophobicity. In addition, incorporating NPs, followed by a subsequent modification process, can further improve the superhydrophobic (SHP) nature. In a study, TiO_2_ nanoparticles were anchored on polyurethane nanofibers (PU-NFs), followed by PDMS application, which resulted in SHP and UV-resistant nanofiber composite membranes. TiO_2_-NPs provided UV protection and also induced surface roughness, offering SHP qualities [38]. In a similar way, Shan Jiang et al. prepared electrospun nanofibrous PVDF membranes containing 3% SiO_2_ with a WCA of 150.0°, compared to a bare nanofibrous membrane with 138.5° [39]. In another study, the surface energy of the produced PVDF composite nanofibrous membrane that contained SiO_2_ nanoparticles (SiO_2_-NPs) was decreased by silanizing it with fluoroalkyl silane after an acid pre-treatment [40]. Another study used fluorinated PU, which had perfluoro alkane segments, and integrated the SiO_2_-NPs into SHP nanofiber membranes, obtaining an excellent WCA (165°) and oil contact angle (151°) [41].

Although the above-mentioned research provided the surface with excellent water resistance, a facile, eco-friendly, and less expensive approach is required to fabricate water-resistive surfaces, excluding toxic chemicals. In addition, for the practical use of these membranes, adequate mechano-chemical stability and excellent robustness against mechanical deformation, such as strain, abrasion, and bending, are critical parameters.

This research produced a multimodal superhydrophobic nanofibrous membrane, exhibiting good permeability and mechano-chemical stability for water/oil and self-cleaning purposes. In the first phase, the SHP SiO_2_-NPs were synthesized, achieving bubbled-shaped roughness on a nano level. Then, the simultaneous electrospinning and electrospraying process fabricated a textured polyurethane nanofibrous membrane that contained SiO_2_-NPs (SiO_2_@PU-NFs). The SiO_2_@PU-NFs membrane structure was modulated through various concentrations of PU and SiO_2_-NPs. The surface SiO_2_-NPs of the PU-NFs, through the electrospraying techniques, provided critical roughness and a hierarchical structure. In addition, the excellent SHP characteristics under mechanical deformation (abrasion, bending, and stretching) demonstrated improved SiO_2_-NPs adhesion on the fiber’s surface. This novel superhydrophobic nanofibrous membrane can be used commercially for self-cleaning, water/oil separation, and water resistance breathable fabrics because of its excellent durability against mechanical stresses and harsh environments.

## 2. Materials and Methods

### 2.1. Materials

Cetyltrimethylammonium bromide (CTAB, Mw= 364.45 g/mol), sodium hydroxide (NaOH, Mw = 39.997 g/mol), and Tetraethylorthosilicate (TEOS), used for the synthesis of hierarchical nano-rough SiO_2_-NPs, were purchased from Sigma-Aldrih, Hamburg, Germony. Polydimethylsiloxane (PDMS, Sylgard 184, viscosity 5100 cp), containing prepolymer and crosslinker, was acquired from the Dow Corning Corporation, Midland Texas USA, to functionalize SiO_2_-NPs. Pellets of thermoplastic polyurethane (TPU) of the grade Elastollan*^®^* 1185A (Mw = 75,000 g/mol) were obtained from BASF in Germany. The TPU pellets had a specific gravity of 1.12 g/cm^3^, a glass transition temperature of −38 °C, and a melt flow index of 10–20 g/10 min (measured at 190 °C with a load of 8.7 kg). N,N-dimethylformamide (DMF) (Mw = 73.09 g/mol, density = 0.994 g/cc, purity 99%), sodium hydroxide (NaOH) (Mw = 39.997 g/mol, density = 2.13 g/cm³, purity ≥ 99.99%), ethanol (CH_3_CH_2_OH) (density = 0.789 g/mL, purity 99.8%), xylene, toluene, and n-hexane were purchased from Sigma-Aldrich. All chemicals for the electrospinning of fibers and electrospraying of nanoparticles were used without purification. 

### 2.2. Preparation and Functionalization of Bubble-Structured SiO_2_-NPs

An amount of 2 g of CTAB and 7 mL of 2 M NaOH were added to 23 mL of deionized H_2_O, and the mixture was stirred at 80 °C for approximately 120 min. TEOS (9.3 mL) was swiftly injected into the solution, which turned into white precipitates after 4 min. The solution was then continuously stirred for 150 min. After the completion of the reaction, the prepared particles were washed several times with water and ethanol. Then, the particles were dried in an oven for the complete removal of liquids.

**Figure 1:**
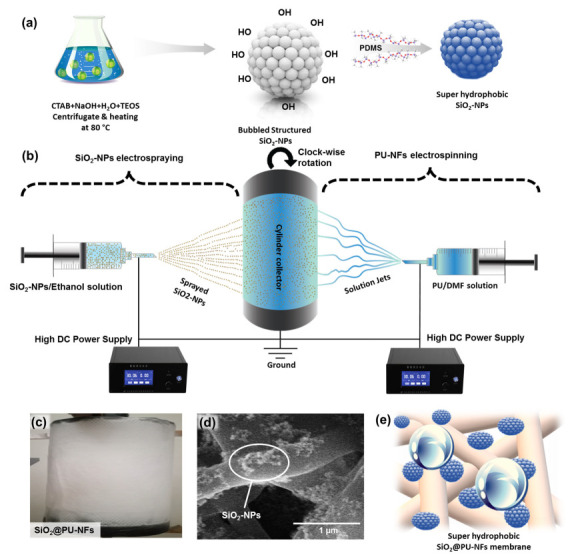
(**a**) Schematic representation of synthesis of bubble-structured SiO_2_-NPs and the chemical modification through PDMS; (**b**) schematic diagram of the simultaneous electrospraying and electrospinning arrangement; (**c**,**d**) photographic view and SEM images of composite SiO_2_@PU-NFs, respectively; (**e**) schematic illustration of water repellency of composite nanofibrous membrane.

PDMS was used to modify the prepared bubble-structured SiO_2_-NPs chemically. In this chemical modification process, 4 to 8 wt% SiO_2_-NPs were dispersed in a combination of PDMS and ethanol solvents. The preparation of these solutions involved a dispersion and ultrasonic treatment process for 2 to 3 h. Then, the solution, comprising the SiO_2_-NPs, PDMS, and ethanol, was used for electrospraying. The schematic representation of bubble-structured SiO_2_-NPs synthesis and its chemical modification through PDMS is shown in Figure 1a.

### 2.3. Preparation of Electrospinning Solutions

Homogeneous PU solutions of 16, 18, and 20% by weight concentrations were prepared using a continuous magnetic stirring process at 500 rpm and 55 °C, lasting 16 h. The resulting solutions were then utilized for electrospinning.

### 2.4. Composite Nanofibrous Membrane Synthesis through Electrospinning/Electrospraying

A needle electrospinning apparatus (Linari nanotech) was employed for the PU nanofibers (PU-NFs) preparation, while SiO_2_-NPs were simultaneously electrosprayed on the nanofibrous membrane. This arrangement is shown schematically in Figure 1b. The electrospinning of the PU-NFs was carried out with a 25 kV voltage and 20 cm spinning distance. However, the 15 kV voltage and 20 cm distance were kept for electrospraying. A temperature of 20 °C and 35% relative humidity was maintained throughout the process. A composite nanofibrous membrane was collected on an aluminum foil-covered cylinder rotating at 150 rpm. Figure 1c–e represent the photograph and SEM of the prepared SiO_2_@PU-NFs membrane, with a schematic illustration of the water repellency of the composite membrane.

In the preliminary experimentation phase, the PU solution’s electrospinning optimization was performed in the range of 16 to 20 wt%. Finally, the 18% PU solution, which exhibited the most favorable electrospinning characteristics, was selected for synthesizing a composite nanofibrous membrane. In addition, the electrospraying of 4 to 8 wt% SiO_2_-NPs with PDMS and ethanol was carried out. Consequently, three composite nanofibrous membranes were fabricated, and the optimal experimental parameters were finalized, followed by an evaluation of the functional characteristics.

### 2.5. Characterization

The morphology of the nanofibrous membranes was analyzed using a field emission scanning electron microscope (FE-SEM), FEI NOVA 450. Thin layers of gold were sputtered onto all samples for 120 s using a sputter coater (Quorum 150R, East Sussex, UK). After acquiring the SEM images of each sample, Image J software was used to calculate the average diameter of approximately 100 fibers. Fourier transform infrared spectroscopy (FTIR, PerkinElmer, Waltham, MA, USA) was used to analyze the functional groups of the nanofibrous membrane in the range of 500–4000 cm^−1^. The hydrophobicity was determined by calculating the samples’ WCAs through a contact angle meter (Attension, Theta Lite, Biolin Scientific, Beijing, China). A 5 µL water droplet was dropped onto the nanofibrous membranes’ surface, and the mean WCA was calculated using a minimum of five values.

The WCA stability against the composite nanofibrous membrane bending was determined using the Kawabata bending module (KES-FB2) evaluation system. A nanofibrous membrane, with a 0.5 cm thickness and 5 × 5 cm dimension, was clamped in the jaws and curved to a −2.5 and +2.5 cm^−1^ radius. One hundred bending cycles were repeated to determine the WCA stability of the samples.

The WCA constancy of the samples, under mechanical deformation, such as abrasion resistance, was measured on the Martindale Abrasion Tester (Roaches), following ISO 12947-2. The circular component, measuring 38 mm in diameter, was placed face down on a regular wool abrasive fabric and subjected to a 9.0 kPa force. The sample was rotated one hundred times at the speed of one rotation per second. The impact of the axial strain on the WCA was estimated by stretching the membrane samples at different strain levels, from 0 to 100%, and the durability of the superhydrophobicity was examined up to 100 stretch cycles. In addition, the WCA was compared with and without dipping the nanofibrous membrane into strong alkali and acidic conditions (PH = 1, 3, 5, 7, 9, 11, and 13 with water solvent) for 24 h to determine the hydrophobic chemical resilience of the specimen.

The universal tensile tester (Ametek Lloyd LRX Plus, Berwyn, PA, USA) measured the nanofibrous membrane’s mechanical properties in megapascals (MPa). The size parameters for the samples had dimensions of 60 × 20 mm. Each sample was tested under a load of 50 N at a rate of 10 mm/min. The waterproof performance of the sample was evaluated through hydrostatic pressure (cmH_2_O) with the Hydrostatic Head Tester MO18 SDL Atlas, as per AATCC 127. A sample of a 100 cm^2^ area was subjected to a steady pressure of 10 cmH_2_O per minute.

Air permeability (AP) and water vapor permeability (WVP) tests were used to assess the breathability of the samples. The Air Permeability Tester MO21A SDL Atlas was used to examine the AP. Samples of a 20 cm^2^ area were subjected to 100 Pa pressure, according to the ISO9237 standards. Five samples were tested, and the average value was recorded. The WVP of the samples was measured using a Water Vapor Permeability Tester RF4319 Refond, based on the ASTM E96 standard (the cup method). The tests were performed at a 37 °C temperature with 50% relative humidity and an air velocity of 1 ms^−1^. The WVP was calculated according to Equation (1) below.

(1) $$\mathrm{WVP}=\frac{W_{2}-{\;W}_{1}}{A}\times 24$$

where WVP is measured in g.m^−2^.d^−1^, (W_2_ − W_1_) is the mass difference of the distilled water during testing (g), and A is the area of the sample (m^2^).

The separation efficiency of the water/oil emulsion was calculated by preparing a mixture of water and oil in equal amounts and vacuum filtering it through the sample. The flux and the separation efficiency were determined using Equations (2) and (3), respectively.

(2) $$\mathrm{Flux}\;=\left(\frac{V}{A\times t}\right)$$

(3) $$\mathrm{Sepration}\;\mathrm{efficiecny}=\left(\frac{M_{i}}{M_{f}}\right)\times 100$$

The flux is calculated in Lm^−2^ h^−1^, and the separation efficiency is in %.

Here, V (L) is the permeate water–oil emulsion volume, A (m^2^) is the sample’s active area, and t (h) is the total time in hours of filtration. M_i_ is the total mass of water at the beginning of the experiment, and M_f_ is the amount of water left in the glass after the filtration process. One sample was subjected to ten filtration cycles continuously to check the filtration efficiency.

## 3. Results and Discussion

### 3.1. Surface Morphology and Chemical Composition

**Figure 2:**
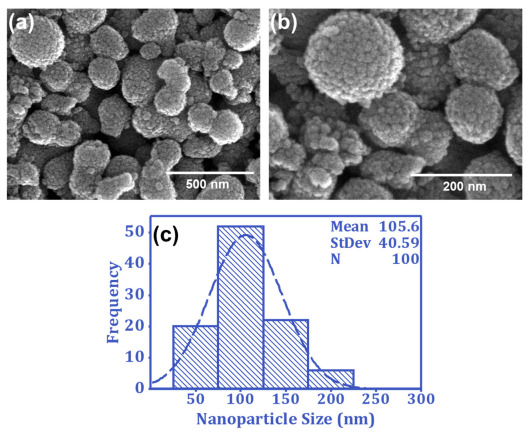
SEM images of bubble-structured SiO_2_-NPs at magnifications (**a**) 50,000 and (**b**) 100,000; (**c**) size distribution of SiO_2_-NPs.

The process and solution parameters control the surface morphology and the SiO_2_-NPs’ size distribution. The surface morphology of the SiO_2_-NPs can be visualized through the SEM images at different magnification levels, as presented in Figure 2a,b. The images demonstrate that SiO_2_-NPs offer dual roughness; the whole nanoparticles create micro-roughness, while the bubble-structured surface of the nanoparticles provides nano-roughness on the fiber’s surface. This dual roughness produces a texture resembling a lotus leaf, with micro- and nano-roughness on the substrate’s surface. The size distribution of the SiO_2_-NPs is presented in Figure 2c. The mean diameter of the SiO_2_-NPs is 105 ± 41 nm, indicating a uniform size distribution.

**Figure 3:**
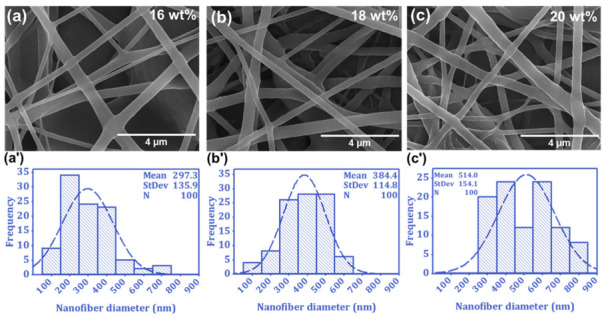
(**a**–**c**) SEM images and (**a′**–**c′**) diameter distribution of PU-NFs at 16, 18, and 20 wt% PU concentration.

**Figure 4:**
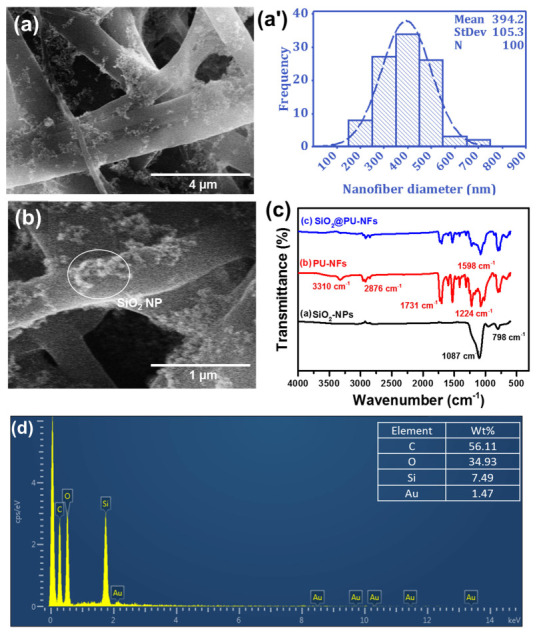
(**a**,**b**) SEM images of f composite SiO_2_@PU-NFs membrane containing SiO_2_-NPs; (**a′**) high-resolution SEM images of composite membrane, revealing the presence of SiO_2_-NPs; (**c**) FTIR spectra of SiO_2_-NPs, PU-NFs, and SiO_2_@PU-NFs; (**d**) elemental analysis of the composite nanofibrous membrane.

The fiber’s diameter and structural shape have significant implications for the performance of the fibers, as they determine properties such as mechanical strength, surface area, and permeability. These factors, in turn, affect the functionality of the electrospun fibers in various applications, such as filtration, tissue engineering, and water–oil separation [42]. Therefore, FE-SEM analysis was employed to evaluate the impact of the solution parameters on the surface morphology and fiber diameter of the PU-NFs, as presented in Figure 3a–c. In contrast, process parameters, such as applied voltage, flow rate, and spinning distance, were held constant at 25 kV, 1.0 mL/h, and 20 cm, respectively. The results indicated that beadless electrospun fibers were formed with all three polymer concentrations, with mean diameters of 297 ± 136, 384 ± 115, and 514 ± 154 nm of the PU-NFs noted at concentrations of 16, 18, and 20 wt%, respectively. Notably, the concentration of the PU significantly impacts the diameter of the PU-NFs, as the diameter of the fiber is significantly increased when the concentration of the PU polymer increases. This finding is consistent with previous reports that documented an increase in fiber diameter with an increase in polymer concentration [43]. The diameter histograms (Figure 3a′–c′) reveal that electrospun fibers with a narrow diameter distribution are fabricated at an 18 wt% PU concentration, whereas electrospun fibers with a wider diameter distribution are obtained at 16 wt%. Furthermore, coarser fibers are formed at 20 wt% with a wide diameter range. Therefore, the 18 wt.% of PU polymer solution was considered the optimized sample and selected for the electrospun fiber membrane. The microscopic structure of the composite SiO_2_@PU-NFs membrane, composed of 6% SiO_2_-NPs and 18% PU concentration, was studied using SEM, as given in Figure 4a. The electrospraying process was unaffected by the fiber diameter distribution and attributed to the nanoparticle deposition on the surface of the PU-NFs. In addition, it can be seen in the SEM images that the SiO_2_-NPs are deposited throughout the surface of the composite PU-NFs. Figure 4a′ shows a histogram of the fiber diameters’ distribution of the optimized SiO_2_@PU-NFs, composed of 6% SiO_2_-NPs and 18% PU concentration. The mean diameter of the composite electrospun fibers is 394 ± 105 nm, respectively, having a narrow uniform diameter distribution. Moreover, the high-resolution SEM image of the SiO_2_@PU-NFs reveals the presence of nanoparticles on the surface of the electrospun fibers (Figure 4b).

Figure 4c shows the FTIR spectra of pristine PU-NFs, the 6% SiO_2_@PU-NFs membrane, and the spectrum SiO_2_-NPs. In pure SiO_2_-NPs, the two crucial peaks, at 1087 and 780 cm^−1^, are characteristic Si-O-Si and Si-C stretching bands, respectively [44]. The absorption band at 3310 cm^−1^ corresponds to the NH stretching for PU-NFs [45].

At the same time, stretching and other modes of -CH_2_ vibrations are identified by the bands at 2859, 2938, 1464, 1364, and 1294 cm^−1^, respectively. In addition, the absorption bands seen at 1731 and 1100–1248 cm^−1^ are associated with a C=O and O-C-O asymmetric stretching in PU [46]. Moreover, the absorption peak at 1598 cm^−1^ was associated with an aromatic group in the PU-NFs [47]. In composite nanofibrous membranes, prominent and significant changes, such as the appearance of distinct characteristic peaks of nanoparticles in FTIR spectra, reveal the presence of SiO_2_-NPs on the PU-NFs surface. In the SiO_2_@PU-NFs membrane, the sharp peaks around 1068 cm^−1^ corresponds to Si-O-Si stretching.

Figure 4d indicates the chemical composition of the SiO_2_@PU-NFs membrane obtained from EDS spectra. EDS analysis of the sample qualitatively confirms the presence of Si and O elements. The SiO_2_ arrays contain 27.98 wt% of Si. These results are consistent with the composition of the SiO_2_ dispersion used in the electrospraying process.

### 3.2. Water Repellency of the Composite Electrospun Fiber Membrane

**Figure 5:**
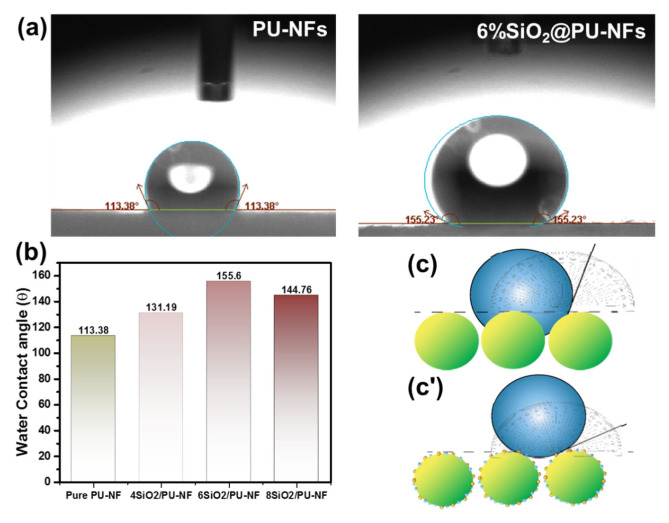
(**a**) WCA of pure PU-NFs and composite SiO_2_@PU-NFs; (**b**) impact of different SiO_2_-NPs concentration on the WCA of SiO_2_@PU-NFs; (**c**,**c′**) schematic illustration of hydrophobic PU-NFs and superhydrophobic and superhydrophobic composite SiO_2_@PU-NFs membrane.

The ability of the composite nanofibrous membrane to oppose water penetration was used to assess its waterproof breathable characteristic [48]. The WCA estimated the superhydrophobicity of the composite nanofibrous membrane of SiO_2_@PU-NFs. As illustrated in Figure 5a, the WCA of the composite SiO_2_@PU-NFs membrane is 155.23° ± 1.96°, which is higher than that of the pristine PU-NFs membranes (113.38° ± 3.9°). This increase in the WCA can be attributed to the fiber’s surface morphology and surface energy, resulting from incorporating SiO_2_ nanoparticles into the PU-NFs composite membrane [49].

The PU-NFs membrane exhibits a low WCA due to its smooth surface morphology, providing a flat surface for water droplets. In contrast, the SiO_2_-NPs enhance the roughness of the SiO_2_@PU-NFs membrane, as explained by the SEM images in Figure 4a,b, resulting in superhydrophobicity. As a result, the WCA of the SiO_2_@PU-NFs membrane is increased by 36.91% compared to the pristine PU-NF membrane. Figure 5b illustrates the impact of different SiO_2_-NPs’ deposition on the composite electrospun fiber membrane, where the highest WCA of 155.6 ± 1.87° was achieved at a 6% SiO_2_-NPs deposition concentration; further increasing the concentration decreased WCA to 144.76 ± 2.31 due to SiO_2_-NPs’ aggregation at higher concentrations. This indicates that incorporating SiO_2_-NPs into the PU-NFs composite membrane not only enhances its surface roughness but also requires the optimization of processing variables for achieving maximum superhydrophobicity.

Figure 5c,c′ visually demonstrates the underlying principle governing the WCA of the pristine PU-NFs membrane and the composite SiO_2_@PU-NFs membrane. Compared to the SiO_2_@PU-NFs membrane, the pure PU-NFs membrane exhibits interfacial tension, causing water droplets to be pulled towards it. On the other hand, the nanoparticles on the modified electrospun fibers’ surface impart nano-roughness, leading to an increased WCA.

### 3.3. Mechano-Chemical Durability Test of Superhydrophobic Membrane

**Figure 6:**
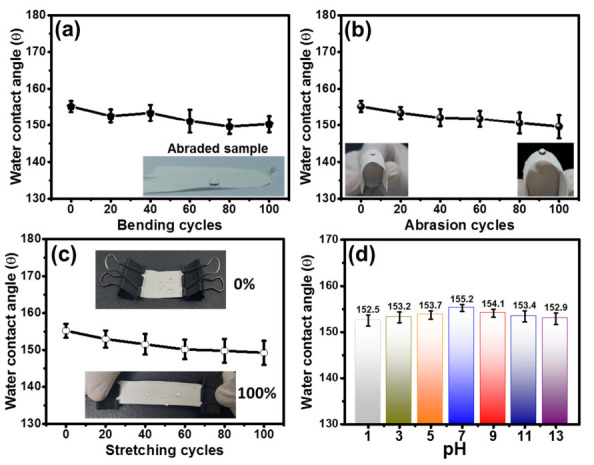
Robustness of the WCA of SiO_2_@PU-NFs membrane against the (**a**) bending cycles, (**b**) abrasion test, (**c**) uniaxial stretching cycles, (**d**) acidic and alkali environmental conditions.

When assessing the effectiveness and lifespan of nanofibrous membranes in practical applications, the robustness of the superhydrophobic surface is a crucial consideration [50,51,52]. The resilience of the WCA of the SiO_2_@PU-NFs membrane is determined at 100 bending cycles, as presented in Figure 6a. The sample was bent to 90° in an upward direction and then moved back to its original position; after this, the specimen was further bent to 90° downward and then moved back to its original position in one complete cycle. The WCAs of the composite membrane remain greater than 150°, even after 100 bending cycles.

The SiO_2_@PU-NFs membrane was also subjected to sandpaper abrasion with a load of 100 g and a transverse distance of 5 cm during a cycle [53]. The influence of the abrasion cycles from 0 to 100 on the WCA is shown in Figure 6b. The WCA was noted after each of the 20 abrasion cycles. The surface of the composite nanofibrous membrane retained its roughness even after 100 abrasion cycles, and no significant decrease in the WCAs (higher than 150°) was observed.

Thus, this confirms the uniform deposition of the nanoparticles on the nanofibrous surface, mainly due to the simultaneous electrospraying and electrospinning processes. Consequently, severe abrasion could not affect the roughness of the composite membrane. This test proves the excellent robustness and stability of the composite membrane against mechanical stresses. In addition to sandpaper abrasion, the resilience of the composite membrane against axial strain was also investigated [54,55]. As demonstrated in Figure 6c, the durability of the sample, after numerous stretching cycles at a strain of 100%, was also evaluated. A slight variation in WCAs was observed, and the composite membrane retains its superhydrophobicity and functionality throughout the stretching cycles. Figure 6d displays the effect of different pH conditions on the WCA of the composite membrane.

The samples were immersed in 1, 3, 5, 7, 9, 11, and 13 pH solutions for 24 h, and then the WCAs were calculated for these samples. The samples retained their superhydrophobic nature after being immersed in strongly acidic and alkaline solutions for 24 h, indicating excellent chemical resistance. Thus, the SiO_2_@PU-NFs membrane has the potential for practical applications, even in severe conditions [56,57].

### 3.4. Mechano-Chemical Durability Test of Superhydrophobic Membrane

**Figure 7:**
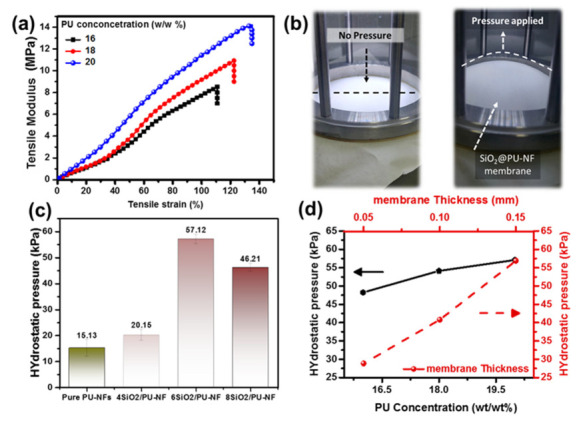
(**a**) Impact of PU concentration on the mechanical performance of the composite nanofibrous membrane; (**b**) photographic view of SiO_2_@PU-NFs membrane under hydrostatic pressure; (**c**) hydrostatic pressure of pristine PU-NFs and SiO_2_PU-NFs membranes; (**d**) impact of the PU concentrations and membrane thickness on the hydrostatic pressure of the composite nanofibrous membrane.

The mechanical performance is a critical characteristic of the electrospun fibers membrane in waterproof, membrane distillation [58,59], and water/oil separation applications. The mechanical properties of the SiO_2_@PU-NFs membranes were examined through the tensile modulus and elongation at break. The impact of the different polymer concentrations on the composite membrane was evaluated through the stress–strain curves shown in Figure 7a. The highest tensile modulus, 14.22 Mpa, is observed at a 20 wt% PU concentration with a tensile strain of 134.85%. Moreover, it can be noted that the composite membrane shows a gradual increase of the tensile modulus and elongation at the break by increasing the polymer concentration. The improved mechanical performance is attributed to the coarser fiber and a defect-free uniform membrane, improving the load-bearing capacity of the composite electrospun fiber. Moreover, an increase in the electrospun fiber diameter enhances the flexibility and elongation at the break of the electrospun fiber membrane, contributing to its good mechanical performance [60,61].

The waterproof capability of the composite membrane was assessed by the hydrostatic pressure [62]. As shown in Figure 7b, the photographs of the composite membrane, before (flat shape) and after pressure are applied (round shape), reveal the stretchability and withstanding of the composite membrane against high hydrostatic pressure. The hydrostatic pressure of the pristine electrospun fiber membrane and modified electrospun fiber membrane with different percentages of the SiO_2_-NPs is given in Figure 7c.

The 6% SiO_2_@PU-NFs membrane exhibits a hydrostatic pressure of 57.12 kPa compared to the 15.13 kPa of pure PU-NFs membrane. Moreover, the hydrostatic pressure for the 4 and 8 wt% is comparatively lower than the 6 wt% of SiO_2_-NPs, which is 20.15 and 46.21 kPa, respectively. Therefore, the effective water repellence of the 6% SiO_2_@PU-NFs is mainly due to the better superhydrophobic nature, attributed to the higher hydrostatic pressure than other nanofibrous membranes.

Figure 7d presents the influence of the PU concentration and membrane thickness on the hydrostatic pressure performance of the composite electrospun fiber membrane. This higher hydrostatic pressure of 57.12 is noted at the 20% polymer concentration and 0.15 mm membrane thickness. This is because of the stronger fibers fabricated at a 20 wt% PU concentration. Moreover, the thicker electrospun fiber contains a significant number of fibers in the cross-section and can bear more water pressure than the thinner electrospun fiber membrane.

### 3.5. Breathability of Superhydrophobic Membrane

**Figure 8:**
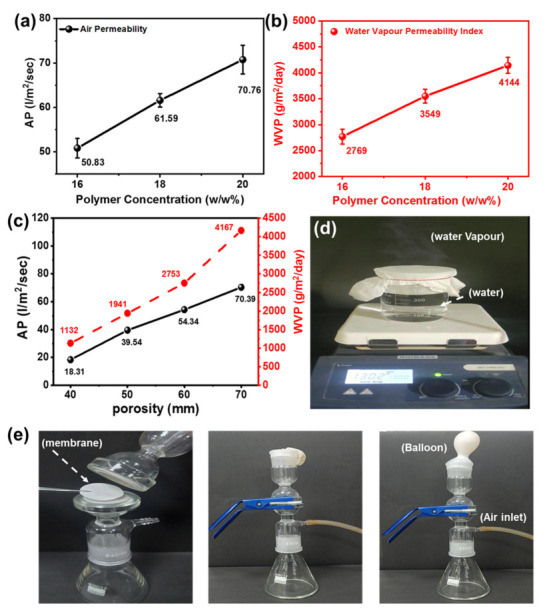
(**a**,**b**) Influence of the polymer concentration on the AP and WVP of the composite PU-NFs membrane, containing 6% SiO_2_-NP; (**c**) impact of the porosity of composite PU-NFs membrane on the air permeability and WVP; (**d**,**e**) self-developed techniques were employed to demonstrate the breathability of the nanofibrous membrane.

The breathability of the developed membrane was evaluated to determine its effectiveness in waterproof breathable end uses [63,64]. Breathability is primarily related to the cumulative effect of AP and WVP. Figure 8a,b illustrates the impact of varying polymer concentrations, specifically within the range of 16 to 18%, on the composite membrane’s AP and WVP, incorporating a constant 6% SiO_2_-NP.

Both AP and WVP increase with the increase in polymer concentration. The membrane’s AP improves by 19.93 L/m^2^/s when the polymer concentration increases from 16 to 20%. Similarly, WVP enhances from 2769 to 4144 g/m^2^/day, increasing the polymer concentration from 16 to 20%. Figure 8c depicts the effect of porosity on both the AP and WVP of composite membranes, which include the 6% SiO_2_NP and PU concentrations, ranging from 16 to 18%. Both AP and WVP increase the porosity with increasing concentrations, where a maximum AP of 70.39 L^2^/m/s and WVP of 4167 g/m^2^/day at a 70% porosity was observed for the composite membrane. Additionally, the optimized nanofibrous membrane, composed of 6% SiO_2_-NPs and 18% PU concentration, was subjected to self-developed techniques to demonstrate its breathability, illustrated in Figure 8d,e. The water vapor generated from the hot water (temperature ~98 °C) can quickly pass from the membrane, as visualized in Figure 8d. Similarly, the air stream, blowing from the running pump, can easily pass through the membrane without damaging the membrane and without inflating the balloon, as shown in Figure 8e. These tests provide evidence of the excellent breathability of the composite membrane.

### 3.6. Self-Cleaning Water/Oil Separation Performance

**Figure 9:**
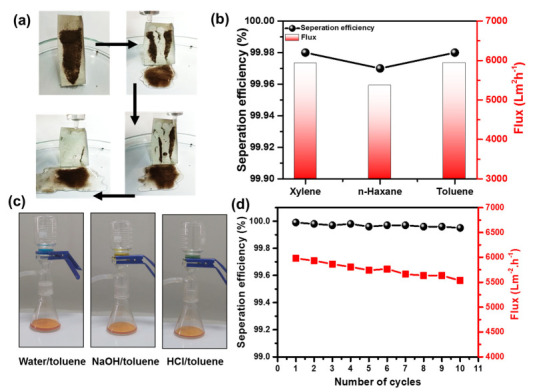
(**a**) Photographic view of the self−cleaning activity of SiO_2_@PU-NFs membrane; (**b**) water/oil separation efficiency and flux, using different Xylene, n−Haxane, and Toulene as non−aqueous liquids; (**c**) photographic view of toluene recovery from other liquids, such as water, alkali, and acid; (**d**) water/oil separation performance of composite nanofibrous membrane under multiple cycles.

The self-cleaning ability of a superhydrophobic surface is an inherent crucial benefit, resulting in potential applications [65,66]. A dirt wipe-off test was conducted to determine the self-cleaning characteristics of the SiO_2_@PU-NFs membrane, as shown in Figure 9a. The membrane was placed in a Petri dish with an angle of less than 10°, and dirt was placed on the membrane. The surface was then exposed to water droplets, which immediately flushed the dirt particulates from the surface of the SiO_2_@PU-NFs membrane upon contact. As a result, the surface was left dry and clean. This exceptional characteristic makes the superhydrophobic composite nanofibrous membrane a promising candidate for various healthcare applications, such as personal protective equipment (PPEs) [67]. In addition to its self-cleaning properties, the SiO_2_@PU-NFs membrane exhibits a significant superhydrophobic character and oleophilic nature, making it an ideal candidate for water/oil separation [68,69]. The membrane’s excellent separation efficiency was demonstrated against various organic liquids, including xylene, n-hexane, and toluene, as indicated in Figure 9b.

The separation efficiency is 99.98, 99.97, and 99.98 against the water/xylene, water/n-hexane, and water/toluene mixers, respectively, whereas the flux is indicated as 5925, 5362, and 5938 L m^−2^ h^−1^, respectively (Figure 9b). Therefore, this demonstrates the SiO_2_@PU-NFs membrane’s superior water/oil separation capability compared to several organic solvent/water mixers. Moreover, the water/oil separation performance of the SiO_2_@PU-NFs membrane was evaluated in harsh environments, including acidic and alkali media, as presented in Figure 9c. In addition to the water/oil separation, the reusability of composite membranes in multiple separation cycles was also assessed. Under successive ten cycles, the separation efficiency remains over 99.9%, and the separation flux is also almost unchanged, more significant than 5500 Lm^−2^ h^−1^, as shown in Figure 9d. Therefore, the SiO_2_@PU-NFs membrane has immense potential for water/oil separation, wastewater treatment, and environmental remediation applications.

## 4. Conclusions

The electrospinning of PU-NFs and simultaneous electrospraying of SiO_2_-NPs synthesized a multipurpose and versatile superhydrophobic composite nanofibrous membrane. The SiO_2_-NPs on the PU membrane produced nano-level roughness. As a result, the composite fibrous membrane displayed excellent superhydrophobicity with a WCA of 155.6°. Furthermore, the as-prepared composite nanofibrous membrane exhibited a uniform fiber diameter distribution with an average diameter of 394 ± 105 nm. The electrospraying process homogeneously deposited the SiO_2_-NPs with a particle diameter of 105 nm onto the fiber’s surface throughout the PU-NFs membrane. As a result, the SiO_2_@PU-NFs membrane exhibited excellent robustness under extreme mechanical conditions. The SiO_2_@PU-NFs membrane maintained its WCA above 150 under 50 cycles of sandpaper abrasion, bending, and uniaxial stretching.

The superhydrophobic composite nanofibrous membrane demonstrated exceptional mechanical properties, such as a tensile modulus of 14.22 Mpa and hydrostatic pressure of 57.12 cmH_2_O. Moreover, the membrane exhibited outstanding breathability characteristics, with an AP of 70.76 mms^−1^ and a WVP of 4144 g·m^2^·d^−1^. In addition, the composite nanofibrous membrane displayed an exceptional water/oil separation efficiency greater than 99%, with a high flux of 5938 L/m^2^/h, even after multiple application cycles. Hence, this superhydrophobic composite nanofibrous membrane holds immense potential for diverse industrial applications, such as water purification and protective textiles, owing to its superior mechanical robustness, breathability, and separation efficiency.

## Data Availability

Data will be provided on request.
